# NATIONAL INSTITUTES OF HEALTH (NIH): SIMPLIFYING THE REVIEW OF RESEARCH PROJECT GRANT APPLICATIONS

**DOI:** 10.1093/geroni/igae098.1800

**Published:** 2024-12-31

**Authors:** Elia Ortenberg, Karen Fingerman, Kira Birditt

**Affiliations:** Chair; Co-Chair; Discussant

## Abstract

The National Institutes of Health (NIH) administers >$45B (FY22) of federal funding for extramural medical research, which supports over 300,000 scientists and 2,500 institutions across the United States. These funds are competitively awarded through a two-stage review process. The first level of review solely evaluates the scientific merit of an application by convening peer reviewers who are experts in the field to adjudicate the potential impact of the proposed research. The second level of review takes into consideration for funding both the scientific impact of a proposal as well as its responsiveness to NIH programmatic objectives. NIH is committed to ensuring that all applications for funding receive fair, independent, expert, and timely scientific reviews so the agency can fund the most promising research. NIH upholds its mission of maintaining high standards in peer review by implementing standardized processes and methodologies across all review panels. NIH also continually focuses on efforts to improve peer review. This session is targeted to both experienced and first-time PIs and reviewers. The goals are to provide a general overview of how applications are reviewed by the Center for Scientific Review (CSR) and discuss important changes that impact grant applications, including NIH’s current initiative to simplify the peer review framework for research project grant applications and CSR’s process for ensuring that study sections change with the science. NIH’s ultimate goal through this effort is to identify the best, most innovative science with the potential to improve human health or advance our scientific understanding.

